# A comparison of temporal artery thermometers with internal blood monitors to measure body temperature during hemodialysis

**DOI:** 10.1186/s12882-018-0938-x

**Published:** 2018-06-14

**Authors:** Meaghan Lunney, Bronwyn Tonelli, Rachel Lewis, Natasha Wiebe, Chandra Thomas, Jennifer MacRae, Marcello Tonelli

**Affiliations:** 1 Department of Community Health Sciences, Cumming School of Medicine, University of Calgary, 3330 Hospital Dr. NW, Calgary, AB T2N 4N1 Canada; 2 Department of Medicine, Cumming School of Medicine, University of Calgary, Calgary, Canada; 3 Department of Medicine, University of Alberta, 8440 112 St. NW., Edmonton, AB T6G 2R7 Canada

**Keywords:** Temperature, Thermometer, Hemodialysis, Bland-Altman

## Abstract

**Background:**

Thermometers that measure core (internal) body temperature are the gold standard for monitoring temperature. Despite that most modern hemodialysis machines are equipped with an internal blood monitor that measures core body temperature, current practice is to use peripheral thermometers. A better understanding of how peripheral thermometers compare with the dialysis machine thermometer may help guide practice.

**Methods:**

The study followed a prospective cross-sectional design. Hemodialysis patients were recruited from 2 sites in Calgary, Alberta (April – June 2017). Body temperatures were obtained from peripheral (temporal artery) and dialysis machine thermometers concurrently. Paired t-tests, Bland-Altman plots, and quantile-quantile plots were used to compare measurements from the two devices and to explore potential factors affecting temperature in hemodialysis patients.

**Results:**

The mean body temperature of 94 hemodialysis patients measured using the temporal artery thermometer (36.7 °C) was significantly different than the dialysis machine thermometer (36.4 °C); *p* < 0.001. The mean difference (0.27 °C) appeared to be consistent across average temperature (range: 35.8–37.3 °C).

**Conclusions:**

Temperature measured by the temporal artery thermometer was statistically and clinically higher than that measured by the dialysis machine thermometer. Using the dialysis machine to monitor body temperature may result in more accurate readings and is likely to reduce the purchasing and maintenance costs associated with manual temperature readings, as well as easing the workload for dialysis staff.

## Background

Measuring pre-dialysis body temperature is a routine part of care in many hemodialysis (HD) units. Although the gold standard for body temperature measurement is the temperature of central (core) blood, measurements of this parameter are not available in most clinical settings, and so temperature is typically measured at other sites, such as oral, axillary, tympanic, or temporal artery.

Modern HD machines are often equipped with an internal blood temperature monitor that can display core temperature readings. Despite this, common practice in many units is to record manual temperatures using peripheral thermometers. This practice is potentially unnecessary and may introduce error, given the known inaccuracy of peripheral temperature measurement [1].

In this study, we compared body temperature measured by HD machine thermometers with those measured by temporal artery (TA) thermometers. Our secondary objective was to assess the feasibility of replacing the currently used TA method with dialysis machine (DM) thermometers.

## Methods

### Study design

This prospective cross-sectional study is presented according to the STROBE guidelines for cross-sectional studies [2]. This study was approved by the University of Calgary Conjoint Ethics Board.

### Eligibility criteria

All adult (≥ 18 years) patients with a diagnosis of end-stage renal disease, currently undergoing hemodialysis for at least one month at one of the two study sites in Calgary, Alberta, and able to provide informed consent were invited to participate. Patients were recruited between April and June 2017.

### Outcomes

The primary outcome of the study was body temperature (°C). Other outcomes of interest were whether mean temperature varied depending on certain patient characteristics (self-reported gender, age, years on dialysis, diabetes, etc.) and thermometer preference of patients and nurses.

### Data collection

All patients treated in Calgary hemodialysis units are dialyzed using the Fresenius 5008 machine. This machine is equipped with an internal blood monitor that measures arterial temperature to two decimal places, which is transmitted to each unit’s electronic medical record system. Current practice locally is to monitor the patients’ temperature using a TA thermometer (Exergen Corporation, Massachusetts/United States). This device uses infrared technology to measure temporal artery temperature to 1 decimal place. Dialysis nurses and nurses’ aides collected the temperatures at the start of dialysis treatment on a best efforts basis. In some cases, temperature was collected later in the treatment, which was recorded as such. All data was collected during a single dialysis session per patient. Nurses or aides measured body temperature with the TA and DM methods simultaneously. Three measurements were taken for each method, 1 min apart (a total of 6 measurements). Participants were asked whether they preferred to have their temperature measured by the peripheral thermometer or directly by the HD machine. Nurses were also asked to submit anonymous surveys to the research assistants describing their preferences and willingness to use the dialysis machine reading.

### Sample size calculation

A sample size calculation indicated that 90 patients would be required to yield 80% power to demonstrate a 0.3 °C difference between thermometer method pairs, assuming alpha = 0.05. The standard deviation of the difference between methods was assumed to be 1 °C, consistent with prior literature [3–5].

### Statistical methods

The mean difference and the 95% confidence interval (CI) of the two thermometer types (TA - DM) were calculated. In the primary analysis, we used the first pair of temperature measurements from each patient (one temperature using TA and one using DM), regardless of the time at which those measurements were made. A paired t-test was used to test the temperatures of each thermometer type. A Bland-Altman plot [6] was created to assess the level of agreement. Because dialysis treatment might alter body temperature, we also did a sensitivity analysis including only pairs of first temperatures that were obtained during the first 15 min of treatment initiation. We did a second sensitivity analysis that compared the mean value of the three temperatures within the first 15 min of treatment by each method (TA and DM).

Temperatures were compared between groups using t-tests (dichotomous variables) or ANOVA (categorical variables). A quantile-quantile plot was used to compare the distribution of temperature between thermometers.

## Results

### Demographics

In total, 94 patients provided consent and all were included in the analysis (Table 1). Over half (59%) were male, the mean ± SD age was 65 ± 13.6 years, and the median number of years since dialysis initiation was 4 years (Table 1). Body temperature was measured during the first 15 min of the HD treatment in 87 patients (92.6%). Mean temperatures among HD patients were 36.4 °C by the HD machine and 36.7 °C by the temporal artery thermometer.

**Table 1:** Characteristics of hemodialysis patients

	*n* = 94
Age; years (mean (SD))	65 (13.6)
Male: n (%)	59 (62.8)
Diabetes: n (%)	50 (53.2)
Years on dialysis (Median (IQR))^a^	4 (2–7)
Site:	
1: n (%)	44 (47)
2: n (%)	50 (53)
Dialysis Shift^b^	
Morning: n (%)	22 (23)
Afternoon: n (%)	37 (40)
Evening: n (%)	35 (37)

Mean (SD) unless stated otherwise

^a^n = 92

^b^Dialysis treatments are organized into shifts that commence at the following times: morning: 06:30–07:30 h; afternoon: 12:30–13:30 h; evening: 18:00–19:00 h

### Temperatures as measured by temporal artery versus HD machine thermometers

**Fig. 1:**
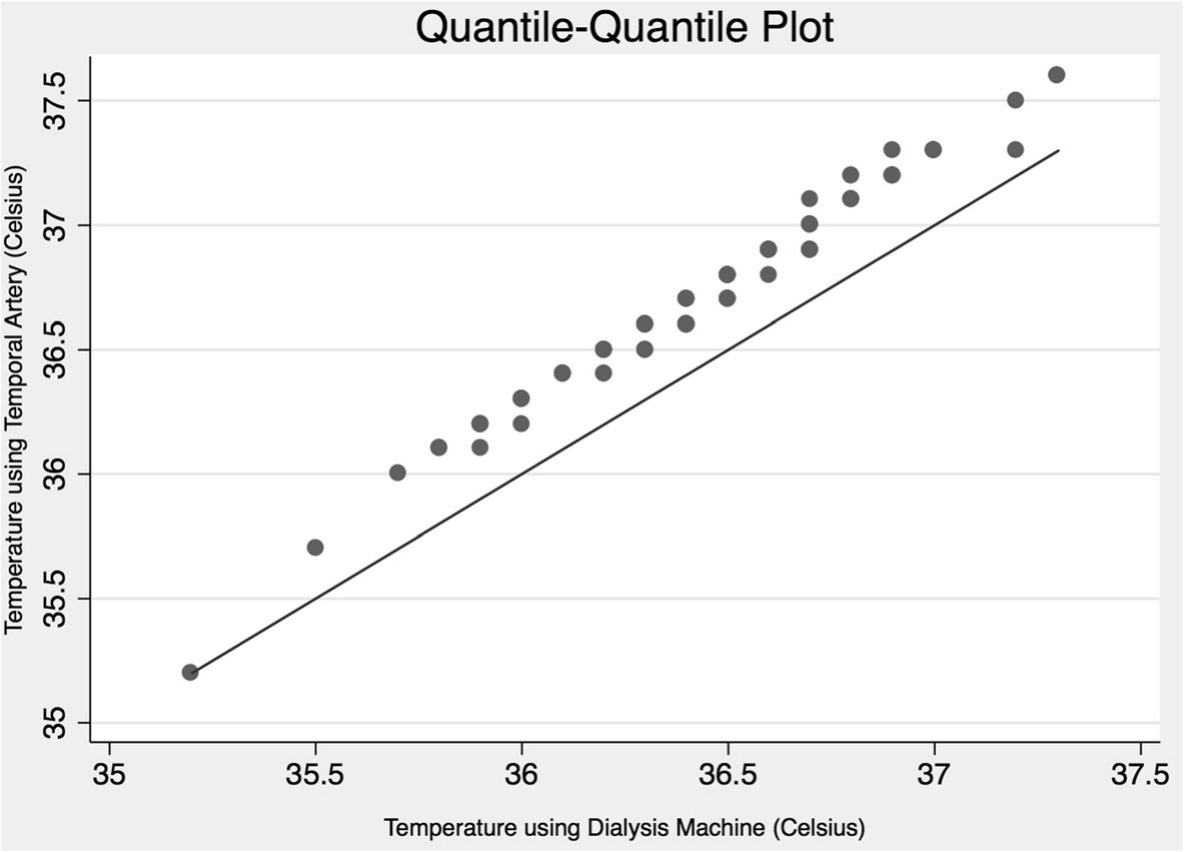
Quantile-quantile (Q-Q) plot comparing the distribution of body temperature of hemodialysis patients, measured by a temporal artery thermometer and an internal blood monitor of the dialysis machine. This plot assesses whether the distribution of temperatures are equal between the two thermometer methods (using the first temperature reading of each device only). As the temporal artery values are all consistently above the reference line, this Q-Q plot suggests a higher temperature when using the temporal artery thermometer, compared to the dialysis machine thermometer. Temperatures measured using the dialysis machine were rounded to single digit

**Fig. 2:**
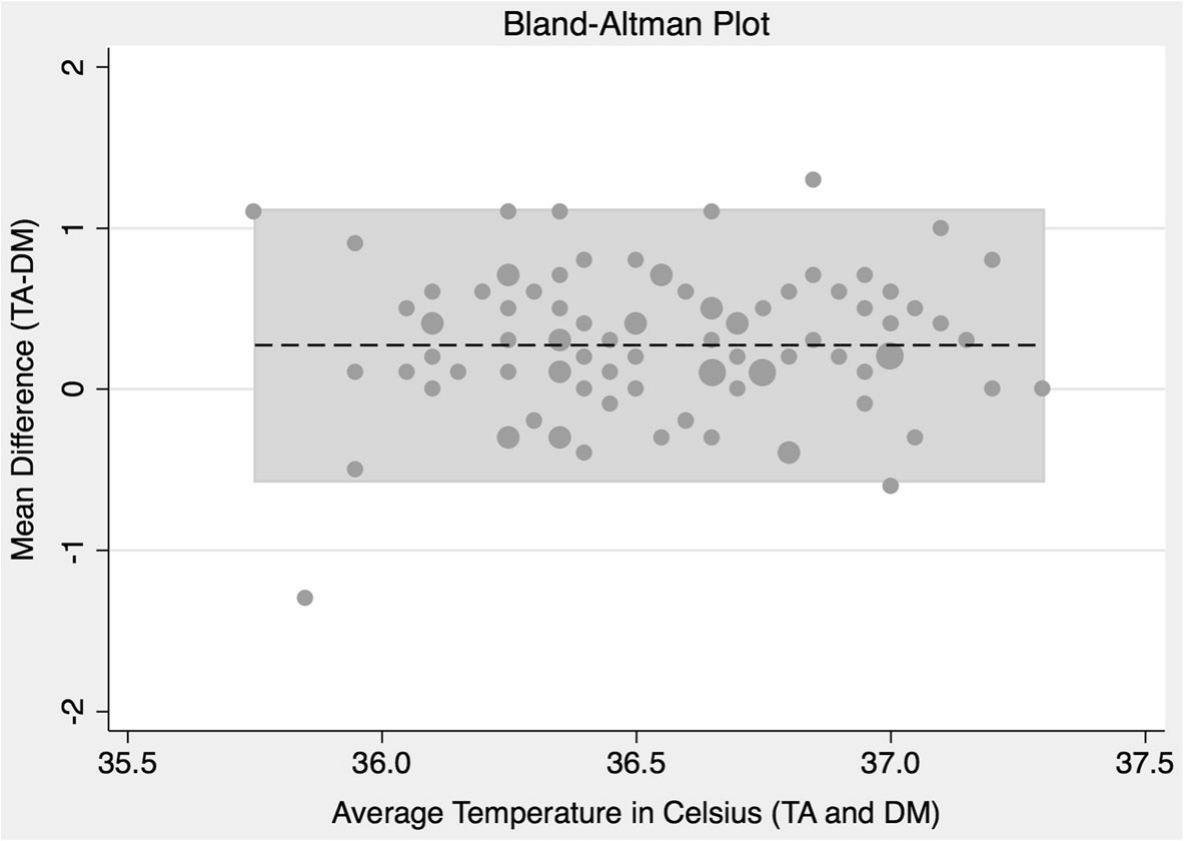
Bland-Altman Plot showing level of agreement between the temporal artery (TA) and dialysis machine (DM) thermometers. Of the 94 data points, 3 (3.2%) are outside the limits of agreement (− 0.57 and 1.12 °C). The mean difference (dotted line) is 0.27 °C and is consistent over the average temperatures obtained in this study. Temperatures measured using the dialysis machine were rounded to single digit. Larger points represent multiple patients with identical values

The distribution of the TA and DM measurements were both normally distributed (Fig. 1). The mean temperature measured by the TA thermometer was 36.7 °C (Table 2), which was significantly different (*p* < 0.001) than the mean DM temperature of 36.42 °C (Fig. 1). The mean difference (95% CI) between the TA and DM temperature was 0.27 °C (0.18–0.37). The Bland-Altman test for agreement showed that 3/94 observations were outside the limits of 95% agreement (Fig. 2). We found that 2/94 (2.1%) patients had temperatures below 36 °C using the TA thermometers, compared to 12 (12.8%) using the dialysis machine thermometer.

**Table 2:** Body temperature of hemodialysis patients measured by the temporal artery thermometer and the internal blood monitor of the dialysis machine for each dataset

Dataset^a^	Thermometer^b^	Mean BT (°C)	95% CI	*p*-value^c^
A	Temporal Artery	36.7	36.6–36.8	< 0.001
	Dialysis Machine	36.42	36.33–36.51	
	Mean Difference	0.27	0.18–0.37	
B	Temporal Artery	36.7	36.6–36.8	< 0.001
	Dialysis Machine	36.42	36.33–36.50	
	Mean Difference	0.27	0.18–0.37	
C	Temporal Artery	36.7	36.6–36.7	< 0.001
	Dialysis Machine	36.49	36.41–36.56	
	Mean Difference	0.21	0.13–0.28	

*BT* body temperature

^a^A: first measurement only, irrespective of time (n = 94); B: first measurement only, within first 15 min of starting treatment (n = 87); C: all measurements (maximum of 3 per thermometer type), within first 15 min of starting treatment (*n* = 87)

^b^Difference = (TA – DM)

^c^Paired t-test comparing the mean temporal artery and dialysis machine temperatures

### Sensitivity analysis

Results were similar in the sensitivity analysis that compared only the first pairs of temperature measurement within the first 15 min of treatment (36.7 °C and 36.42 °C for TA and DM respectively, *p* < 0.001) (Table 2). Results were also similar in the second sensitivity analysis, which compared the mean of the first three measurements within the first 15 min of treatment (36.7 °C and 36.49 °C respectively, *p* < 0.001) (Table 2).

### Preferences of patients and nurses

The large majority (95%) of patients did not express a preference about the thermometer type. Of the 12 nurses that completed a survey, 75% (*n* = 9) preferred using the dialysis machine instead of the forehead thermometer to measure patient body temperature. Reasons given for this included increased convenience and accuracy, reduced workload, and less perceived risk of transmitting infection between patients using the TA thermometer.

### Epidemiology of body temperature among HD patients

There was no evidence that body temperature among HD patients statistically differed by the time of day. For example, median temperatures as measured by the HD machine were 36.24 °C during shift 1, 36.44 °C during shift 2, and 36.50 °C during shift 3) (*p* = 0.53). We also found no significant differences in median body temperature by gender (36.40 °C in males, 36.44 °C females; *p* = 0.68), age (36.49 °C in patients less than 65 and 36.36 in patients ≥65 years, *p* = 0.15), or years on dialysis (36.43 °C if < 6 years, 36.40 °C if ≥6 years, *p* = 0.72). The temperature of patients with diabetes (36.33 °C) was significantly lower than those without (36.52 °C) *p* = 0.02.

## Discussion

We found a statistically significant difference between body temperatures measured using temporal artery thermometers and core blood temperature measured by the dialysis machines. The mean difference between the methods was 0.27 °C, with the TA device reporting higher temperatures than the DM method. If one considers the DM method to be the gold standard, the limits of agreement (1.69 °C) exceed the recommended clinically acceptable difference of 0.5 [7], suggesting that the TA device may not be ideal for routine clinical use, given that a superior measurement is readily available.

To our knowledge, this is the first study comparing methods of temperature measurement in HD patients. Studies done in other populations have also reported a difference between temperatures measured using peripheral thermometers compared to those measuring core temperatures [1], [8–10]. A meta-analysis of mean differences in afebrile patients reported that temporal artery thermometers were 0.07 °C higher than the reference core temperature [8]. However, this analysis included children and adults and a variety of core thermometer types, which could explain the lower discrepancy than found in our study. Interestingly, the meta-analysis found that TA thermometers underestimated temperatures among febrile patients (mean difference of − 0.19 °C). Taken together with our findings (overestimation of temperature among HD patients), these findings suggest that the temperatures obtained using TA thermometers may bias towards the mean (“normal”) temperature as seen in healthy populations. However, since all of the HD patients we studied were afebrile, this suggestion is speculative.

We found no association between body temperature and factors such as age, gender, years on dialysis, or time of day. This finding contrasts with previous research that showed a significant difference by gender [11] and time of day [12] in healthy individuals. Possible explanations for the discrepant finding could be related to kidney failure or because the participants in this study were older (IQR: 56–75 years). Patients with diabetes had significantly lower body temperature than those without, although the magnitude of the difference was small.

The findings from this study have potential implications for clinical practice. Considering core temperature as the gold standard, the temperatures measured by the dialysis machine thermometer were statistically different from those measured by the temporal artery thermometers. Although the magnitude of the difference was small, it might be clinically relevant in some scenarios. In addition, recording temperature from the dialysis machine may save small amounts of staff time compared to measuring it with a thermometer, especially if the data can be captured automatically by linkage between the dialysis machine and an electronic health record. In our study, although patients did not have a preference, most nurses preferred to measure temperature using the dialysis machine.

While this study achieved 80% power, included a representative HD population, and collected temperature measurements using two clinically relevant methods, it also has potential limitations. First, different nurses were involved in the data collection. Due to possible varying techniques, this could impact the precision of the temporal artery thermometers. However, this reflects real-world clinical practice and therefore is appropriate for understanding the practical implications of this study. Secondly, while we aimed to always obtain the pair of study measurements within the first 15 min of treatment initiation, this was not possible in 7 of 94 patients. Results were similar in our sensitivity analysis (done only in patients whose measurement was made within the first 15 min of treatment), suggesting that inclusion of these 7 patients did not affect our results. One could argue that a more stringent requirement (e.g. making all measurements within the first 30 s of dialysis) would have been preferable. However, although dialysis treatment may change true body temperature, there is no strong reason to think that it should differentially affect the temperature as measured by one technique vs. another. Nonetheless, this is a potential limitation of our study. Thirdly, these findings may not be generalizable to patients with significant access recirculation, which may affect the accuracy of core temperature measurement by the DM method. Furthermore, dialysate temperatures were not monitored in this study, which may have potential to influence the differences between the two thermometer types. However, these are unlikely to have affected our main conclusions. Finally, no febrile patients were included in this study and therefore the diagnostic performance of the two methods for detecting fever could not be compared.

## Conclusions

The mean body temperature of hemodialysis patients as measured by a temporal artery thermometer was 0.27 °C higher than the mean core temperature measured by the dialysis machine. As core temperature is the gold standard, using the dialysis machine to measure body temperature in HD patients rather than an external thermometer may result in slightly greater accuracy while possibly also lowering staff workload.
